# Contributions to the systematics of New World macro-moths III

**DOI:** 10.3897/zookeys.149.2383

**Published:** 2011-11-24

**Authors:** B. Christian Schmidt, J. Donald Lafontaine

**Affiliations:** 1 Canadian Food Inspection Agency, Canadian National Collection of Insects, Arachnids and Nematodes, K.W. Neatby Bldg., 960 Carling Ave., Ottawa, ON, Canada K1A 0C6; 2 Canadian National Collection of Insects, Arachnids, and Nematodes, Biodiversity Program, Agriculture and Agri-Food Canada, KW Neatby Bldg., C.E.F., Ottawa, Ontario, Canada K1A 0C6

This special issue of ZooKeys, “Contributions to the systematics of New World macro-moths III” is the third volume in this series. The series was initiated in May 2009 (ZooKeys # 9), with the second volume published in March 2010 (ZooKeys # 39) (Schmidt and Lafontaine 2009, 2010). Fourteen authors contributed 13 manuscripts for this volume, covering taxa in the Noctuidae, Erebidae, Notodontidae, Geometridae and Crambidae. New taxa are described from Argentina, Bolivia, Canada, Chile, Costa Rica, Peru and United States. Taxonomic changes include the description of two new genera, eight new species, and a new subspecies. Also, 47 new or revised synonyms, six new or revised statuses, and 19 new or revised generic combinations are proposed herein.

Since its inception in 2009, the “Contributions” series collectively includes 37 taxonomic publications by 24 authors, containing 51 new species descriptions, 6 new genera, 119 new or revised synonymies, 22 new or revised statuses, and 143 new or revised generic combinations. Geographic coverage has focused on the North American fauna (Canada, United States and Mexico), but taxa from Central and South America are also covered. For reference, we include below the links to the two previous “Contributions …” in ZooKeys.

Authors interesting in contribution to future “Contributions …” are encouraged to contact us.

## Contents of previous volumes

For reference, we include below links to the two previous “Contributions …” in ZooKeys.

### ZooKyes 9 (2009) Contributions to the systematics of New World macro-moths

1	Schmidt BC, Lafontaine JD (2009) Contributions to the Systematics of New World Macro-Moths. In: Schmidt BC, Lafontaine JD (Eds) Contributions to the Systematics of New World Macro-Moths. ZooKeys 9: 1–2. doi: 10.3897/zookeys.9.183

3	Sullivan JB (2009) A new species of *Rivula* Guenée (Lepidoptera, Noctuidae) from southeastern United States. In: Schmidt BC, Lafontaine JD (Eds) Contributions to the Systematics of New World Macro-Moths. ZooKeys 9: 3–10. doi: 10.3897/zookeys.9.185

11	Brou Jr VA, Lafontaine JD (2009) A new species of *Lithophane* Hbn. (Lepidoptera, Noctuidae, Xyleninae) from southeastern United States. In: Schmidt BC, Lafontaine JD (Eds) Contributions to the Systematics of New World Macro-Moths. ZooKeys 9: 11–20. doi: 10.3897/zookeys.9.158

21	Walsh B (2009) *Lithophane leeae* (Lepidoptera, Noctuidae, Xyleninae), a striking new species from southeastern Arizona. In: Schmidt BC, Lafontaine JD (Eds) Contributions to the Systematics of New World Macro-Moths. ZooKeys 9: 21–26. doi: 10.3897/zookeys.9.184

27	Ferris CD, Lafontaine JD (2009) Review of the *Acontia areli* group with descriptions of three new species (Lepidoptera, Noctuidae, Acontiinae). In: Schmidt BC, Lafontaine JD (Eds) Contributions to the Systematics of New World Macro-Moths. ZooKeys 9: 27–46. doi: 10.3897/zookeys.9.180

47	Metzler EH, Bustos D, Forbes GS (2009) The Lepidoptera of White Sands National Monument, Otero County, New Mexico, USA 1. Two new species of *Noctuidae* (Lepidoptera, Noctuinae, Agrotini). In: Schmidt BC, Lafontaine JD (Eds) Contributions to the Systematics of New World Macro-Moths. ZooKeys 9: 47–62. doi: 10.3897/zookeys.9.182

63	Schmidt BC (2009) Revision of the “*Aemilia*” *ambigua* (Strecker) species-group (Noctuidae, Arctiinae). In: Schmidt BC, Lafontaine JD (Eds) Contributions to the Systematics of New World Macro-Moths. ZooKeys 9: 63–78. doi: 10.3897/zookeys.9.149

79	Schmidt BC, Macaulay D (2009) A new species of *Dodia* Dyar (Noctuidae, Arctiinae) from central Canada. In: Schmidt BC, Lafontaine JD (Eds) Contributions to the Systematics of New World Macro-Moths. ZooKeys 9: 79–88. doi: 10.3897/zookeys.9.150

89	Schmidt BC (2009) A new genus and two new species of arctiine tiger moth (Noctuidae, Arctiinae, Arctiini) from Costa Rica. In: Schmidt BC, Lafontaine JD (Eds) Contributions to the Systematics of New World Macro-Moths. ZooKeys 9: 89–96. doi: 10.3897/zookeys.9.151

97	Anweiler GG (2009) Revision of the New World *Panthea* Hübner (Lepidoptera, Noctuidae) with descriptions of 5 new species and 2 new subspecies. In: Schmidt BC, Lafontaine JD (Eds) Contributions to the Systematics of New World Macro-Moths. ZooKeys 9: 97–134. doi: 10.3897/zookeys.9.157

### ZooKeys 39 (2010) Contributions to the systematics of New World macro-moths II

1	Schmidt BC, Lafontaine JD (2010) Contributions to the systematics of New World macro-moths II. In: Schmidt BC, Lafontaine JD (Eds) Contributions to the systematics of New World macro-moths II. ZooKeys 39: 1–2. doi: 10.3897/zookeys.39.442

3	Lafontaine JD, Walsh JB (2010) A review of the subfamily Anobinae with the description of a new species of *Baniana* Walker from North and Central America (Lepidoptera, Erebidae, Anobinae). In: Schmidt BC, Lafontaine JD (Eds) Contributions to the systematics of New World macro-moths II. ZooKeys 39: 3–11. doi: 10.3897/zookeys.39.428

13	Hawks DC (2010) Review of the *Catocala delilah* species complex (Lepidoptera, Erebidae). In: Schmidt BC, Lafontaine JD (Eds) Contributions to the systematics of New World macro-moths II. ZooKeys 39: 13–35. doi: 10.3897/zookeys.39.439

37	Gall LF, Hawks DC (2010) Systematics of moths in the genus *Catocala* (Lepidoptera, Erebidae) IV. Nomenclatorial stabilization of the Nearctic fauna, with a revised synonymic check list. In: Schmidt BC, Lafontaine JD (Eds) Contributions to the systematics of New World macro-moths II. ZooKeys 39: 37–83. doi: 10.3897/zookeys.39.425

85	Sullivan JB (2010) A new genus and species for *Dysgonia* (Lepidoptera, Erebidae, Erebinae) from Southeastern United States. In: Schmidt BC, Lafontaine JD (Eds) Contributions to the systematics of New World macro-moths II. ZooKeys 39: 85–97. doi: 10.3897/zookeys.39.434

99	Schmidt BC (2010) Taxonomic reassessment of *Zale lunifera* (Hübner) (Erebidae, Erebinae). In: Schmidt BC, Lafontaine JD (Eds) Contributions to the systematics of New World macro-moths II. ZooKeys 39: 99–106. doi: 10.3897/zookeys.39.431

107	Wagner DL, Binns S (2010) Larva and pupa of *Amyna axis* (Guenée, 1852) and affirmation of its taxonomic placement in Bagisarinae (Lepidoptera, Noctuidae). In: Schmidt BC, Lafontaine JD (Eds) Contributions to the systematics of New World macro-moths II. ZooKeys 39: 107–116. doi: 10.3897/zookeys.39.435

117	Ferris CD, Lafontaine JD (2010) Review of the North American species of *Marimatha* Walker with descriptions of three new species (Lepidoptera, Noctuidae, Eustrotiinae) and the description of *Pseudomarimatha flava* (Noctuidae, Noctuinae, Elaphriini), a new genus and species confused with *Marimatha*. In: Schmidt BC, Lafontaine JD (Eds) Contributions to the systematics of New World macro-moths II. ZooKeys 39: 117–135. doi: 10.3897/zookeys.39.424

137	Lafontaine JD, Poole RW (2010) Review of the New World genera of the subfamily Acontiinae (Lepidoptera, Noctuidae). In: Schmidt BC, Lafontaine JD (Eds) Contributions to the systematics of New World macro-moths II. ZooKeys 39: 137–160. doi: 10.3897/zookeys.39.427

161	Schmidt BC, Anweiler GG (2010) The North American species of *Charadra* Walker, with a revision of the *Charadra pata* (Druce) group (Noctuidae, Pantheinae) In: Schmidt BC, Lafontaine JD (Eds) Contributions to the systematics of New World macro-moths II. ZooKeys 39: 161–181. doi: 10.3897/zookeys.39.432

183	Handfield L, Handfield D (2010) *Cucullia umbratica* (Lepidoptera, Noctuidae), a new European noctuid in North America. In: Schmidt BC, Lafontaine JD (Eds) Contributions to the systematics of New World macro-moths II. ZooKeys 39: 183–186. doi: 10.3897/zookeys.39.426

187	Lafontaine JD, Walsh JB, Holland RW (2010) A revision of the genus *Bryolymnia* Hampson in North America with descriptions of three new species (Lepidoptera, Noctuidae, Noctuinae, Elaphriini). In: Schmidt BC, Lafontaine JD (Eds) Contributions to the systematics of New World macro-moths II. ZooKeys 39: 187–204. doi: 10.3897/zookeys.39.437

205	Schmidt BC (2010) Review of the Nearctic species of *Enargia* Hübner, [1821] (Noctuidae, Noctuinae, Xylenini). In: Schmidt BC, Lafontaine JD (Eds) Contributions to the systematics of New World macro-moths II. ZooKeys 39: 205–223. doi: 10.3897/zookeys.39.429

225	Lafontaine JD, Ferris CD, Walsh JB (2010) A revision of the genus *Hypotrix* Guenée in North America with descriptions of four new species and a new genus (Lepidoptera, Noctuidae, Noctuinae, Eriopygini) In: Schmidt BC, Lafontaine JD (Eds) Contributions to the systematics of New World macro-moths II. ZooKeys 39: 225–253. doi: 10.3897/zookeys.39.438

255	Lafontaine JD, Troubridge JT (2010) Two new species of the *Euxoa westermanni* species-group from Canada (Lepidoptera, Noctuidae, Noctuinae). In: Schmidt BC, Lafontaine JD (Eds) Contributions to the systematics of New World macro-moths II. ZooKeys 39: 255–262. doi: 10.3897/zookeys.39.436

263	Sullivan JB (2010) New species of the Neotropical genus *Campatonema* Jones (Geometridae, Ennominae) with the first description of the female. In: Schmidt BC, Lafontaine JD (Eds) Contributions to the systematics of New World macro-moths II. ZooKeys 39: 263–272. doi: 10.3897/zookeys.39.433
